# Effects of increased alcohol availability during adolescence on the risk of all‐cause and cause‐specific disability pension: a natural experiment

**DOI:** 10.1111/add.13750

**Published:** 2017-02-07

**Authors:** Emelie Thern, Jeroen de Munter, Tomas Hemmingsson, George Davey Smith, Mats Ramstedt, Per Tynelius, Finn Rasmussen

**Affiliations:** ^1^ Child and Adolescent Public Health Epidemiology Unit, Department of Public Health SciencesKarolinska InstitutetStockholmSweden; ^2^ Unit of Occupational MedicineInstitute of Environmental Medicine, Karolinska InstitutetStockholmSweden; ^3^ Centre for Social Research on Alcohol and DrugsStockholm UniversityStockholmSweden; ^4^ MRC Integrative Epidemiology Unit (IEU)University of Bristol, School of Social and Community MedicineBristolUK; ^5^ The Swedish Council for Information on Alcohol and Other Drugs (CAN)StockholmSweden; ^6^ Centre for Epidemiology and Community MedicineStockholm County Council, Health Care ServicesStockholmSweden

**Keywords:** Adolescent alcohol consumption, alcohol availability, alcohol policy, disability pension, natural experiment, Sweden

## Abstract

**Aim:**

To test if being exposed to increased alcohol availability during adolescence is associated with an increased risk of receiving disability pension due to all‐cause, alcohol use disorders and mental disorders.

**Design:**

Register‐based population‐based study using a natural experiment setting, the alcohol policy change in Sweden (1967–68), with increased access to strong beer in a narrow time window and geographical area. The individuals exposed to the policy change were compared with non‐exposed individuals living in the rest of Sweden, excluding a border area.

**Setting:**

Sweden.

**Participants:**

A total of 518 810 individuals (70 761 in the intervention group; 448 049 in the control group) born 1948–1953, aged 14–20 years during the policy change.

**Measurements:**

Date and diagnosis of the outcome variable of disability pension due to all‐cause, alcohol use disorders and mental disorders were obtained from the Swedish National Social Insurance Agency database from 1971 to 2013. Individual and family level socio‐demographic and health‐related covariates, as well as a regional level covariate, were included.

**Findings:**

Compared with the control group, adolescents exposed to the alcohol policy change were at an increased risk of receiving disability pension due to all‐causes [hazard ratio (HR) = 1.09, 95% confidence interval (CI) = 1.07–1.11], alcohol use disorders (HR = 1.17, 95% CI = 1.05–1.30) and mental disorders (HR = 1.19, 95% CI = 1.15–1.23).

**Conclusion:**

In Sweden, a natural experiment with a 43‐year follow‐up suggests that exposure to increased alcohol availability during adolescence is associated with an increased risk of receiving a disability pension due to all‐cause, alcohol use disorder and mental disorder diagnoses.

## Introduction

Evidence suggests that increased alcohol availability is associated with increased alcohol consumption, which in turn increases alcohol‐related harm on a population level 1, 2, 3, 4. Previous research, using a natural experiment setting of a policy change, has investigated the short‐ and long‐term effects of changing the legal age limit for alcohol purchases on alcohol consumption and alcohol‐related harm 5, 6, 7, 8, 9, 10. One area where research is scarce, but potential costs are high, concerns the association between increased alcohol availability during adolescence and the risk of disability pension.

In Sweden, disability pension is granted to secure the income of persons with permanently impaired ability to work due to injury or disease. All citizens (irrespective of employment status) from the age of 19–64 years (from age 16 prior to 2003) are entitled to disability pension if they have a medically confirmed disease or injury that impairs their working capacity permanently by at least 25% (50% prior to 1993) 11. In Sweden, disability pension beneficiaries permanently leave the labour market prematurely, either fully or partially. In 1977 there was a change in the Swedish legislation, allowing alcohol abuse to be a disability pension diagnosis 12, 13.

Adolescence appears to be a sensitive period in life with regard to alcohol exposure 14. Several studies have shown that high alcohol consumption during adolescence is associated with high alcohol consumption, alcohol dependence, more mental health and social problems in adulthood 15, 16, 17, 18. Previous research on self‐reported alcohol consumption have found that risk use of alcohol during conscription was associated positively with disability pension due to all‐cause 19, alcohol‐related diagnosis and drug abuse 20, but not associated with disability pension due to a psychosis diagnosis or other psychiatric diagnoses 20, 21. Conversely, a study on females found that a history of alcohol intoxication during adolescence was not associated with increased risk of disability pension 22.

Sweden has a long history of having a restrictive alcohol policy, with a state‐owned retail monopoly enforcing, for example, strict age limits and high prices to restrict alcohol availability 23. In the late 1960s the alcohol policy was liberalized temporarily in two regions in Sweden. Specifically, strong beer became available in grocery stores with a lower age limit (16 years instead of 21 years) 24. A study, using the same alcohol policy experiment as in the current study, found that children of young mothers (below 21 years) exposed *in utero* to this policy change had lower educational attainment, were less likely to be employed and more likely to be dependent on welfare 9.

The effect of policy changes could be different in certain population subgroups. Some research suggests that individuals consuming alcohol already in early adolescence are more vulnerable to the long‐term health consequences compared to individuals who begin consuming alcohol in late adolescence 25, 26, 27, 28. Further, evidence suggests that the consequence of consuming alcohol is more severe among those with lower socio‐economic status 29, 30, but little is known about the potential differential responsiveness to a decrease in the legal age limit for alcohol purchases.

The aim of the current study was to test if being exposed to increased alcohol availability during adolescence (aged 14–20 years) is associated with an increased risk of receiving disability pension due to all‐cause, alcohol use disorders and mental disorders. For this study we will utilize a natural experiment, specifically the 8.5‐month alcohol policy change conducted in Sweden in 1967–68, with an abrupt increase in access to strong beer in a narrow time window and geographical area.

## Method

### Design overview

Cox proportional hazards regression was used to test for an association between increased alcohol availability during adolescence and disability pension using data from a natural experiment setting. We assessed the impact of the alcohol policy change on disability pension due to all‐cause, alcohol use disorders and mental disorders. Models were adjusted for individual‐, family‐ and regional‐level covariates.

### Description of the natural experiment

During 1967 and 1968, an alcohol policy experiment was conducted in Gothenburg and Bohus county and Värmland county (called the intervention area below). Within this geographical area, strong beer containing 4.5–5.6% alcohol by volume became available for purchase in regular grocery stores for individuals aged 16 years or older between 1 November 1967 and 14 July 1968 24. Prior to this, after the experiment, and in the municipalities not involved in the experiment, strong beer was available for purchase only at the Swedish alcohol retail monopoly, Systembolaget, where the age limit was kept at 21 years. In the intervention area there were 26 Systembolag stores selling strong beer prior to the policy change, but during the policy change there were approximately 1530 grocery stores licensed to sell strong beer 9. During the experimental period there was a 10‐fold increase of sales of strong beer compared to the same months during the previous year 24. As there was an increase of reported alcohol‐related problems, especially among young people, the experiment was terminated 6 months earlier than planned initially 24.

### Study population

The study population comprised all non‐adopted individuals born between 1948 and 1953 that were still alive and registered in Sweden 1965 with at least one biological parent identified within the Multi‐Generation Register (*n* = 666 639). To allocate individuals to the intervention or control group, the individual's residence information was extracted from the nearest available Population and Housing Censuses (1965 and 1970). Individuals who did not have information on residence were excluded (*n* = 4500). The intervention group included all individuals living in the intervention area in both 1965 and 1970. Individuals who lived in an area unexposed to the policy change, both in 1965 and 1970, were used as the control group. Individuals who lived in the bordering area (the counties of Halland, Älvsborg, Skaraborg, Örebro and Kopparberg) (*n* = 126 976) were excluded to avoid spill‐over effects. Also, individuals who moved between the areas (*n* = 6141), individuals who emigrated from Sweden (*n* = 796) and died (*n* = 48) between 1965 and 1970 were excluded.

The outcome variable of disability pension was obtained from the Swedish National Social Insurance Agency database (STORE). The register began in 1994; however, the register has backtracked and included all disability pensioners from 1 January 1971 who were still alive in 1994 and receiving disability pension. Consequently, the 9524 individuals (intervention group: 1349, control group: 8175) who died before 1994 were also excluded, as we did not know if these individuals received disability pension. The excluded participants were more likely to be male, born outside Sweden with a lower level of parental socio‐economic status compared to the participants included.

**Figure 1:**
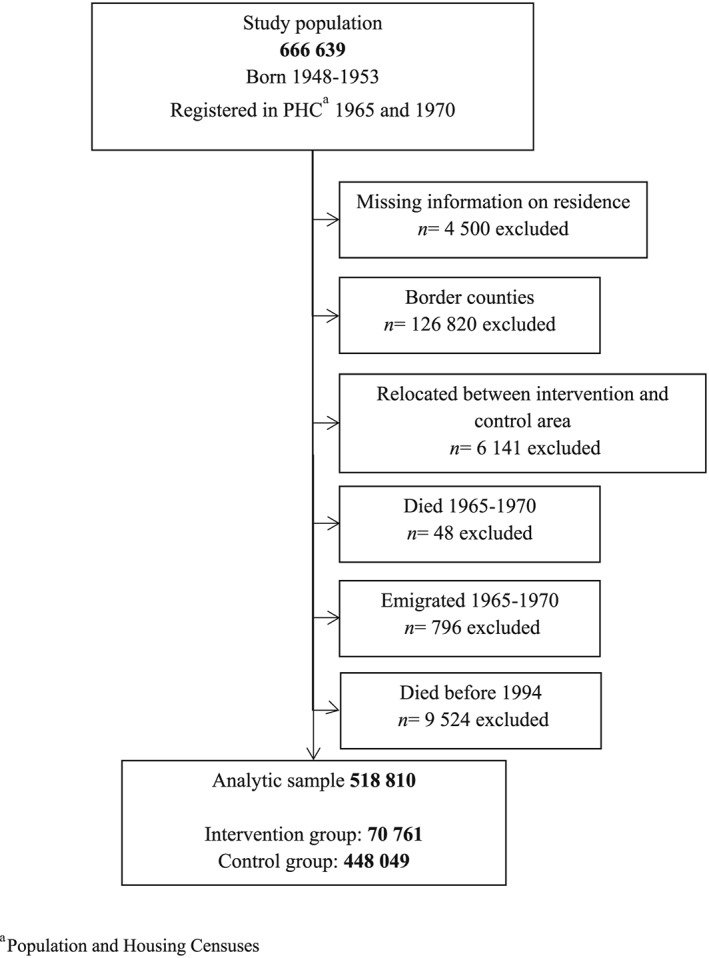
Flow chart describing the selection process of the participants

The final analytical sample consisted of 70 761 individuals in the intervention group and 448 049 in the control group (please see Fig. 1 for more details). The study was approved by the Stockholm Regional Ethical Review Board (Dnr: 2016/112–31).

### Outcome measure: disability pension

Our disability register included the date, the primary diagnosis and occasionally a secondary diagnosis of the disability pension according to the Swedish version of the International Statistical Classification of Disease (ICD) versions 8 (1969–1986), 9 (1987–1996) and 10 (from 1997). The diagnostic groups used were: alcohol use disorders including alcoholic psychoses (ICD‐8 and ICD‐9: 291), alcoholism (ICD‐8 and ICD‐9: 303), misuse of alcohol (ICD‐9: 305) and mental and behavioural disorders due to alcohol abuse (ICD‐10: F10) and mental disorders excluding alcohol use disorders (ICD‐8: 290, 292–302, 304–315; ICD‐9: 290, 292–302, 304, 306–320; ICD‐10: F00‐F09, F20‐F99). First‐, full‐ or part‐time granted disability pension groups were included.

### Covariates

The selection and inclusion of individual‐, family‐ and regional‐level covariates in the analyses was based on prior evidence 21, 31, 32. Individual‐level factors included were sex, year of birth, country of birth and highest level of own attained education. The Multi‐Generation Register (MGR) was used to identify the individual's biological parents. Family‐level covariates included were highest level of parents’ attained education and socio‐economic index (SEI) extracted from the Population and Housing Census 1970. We also included a measure of any of the parents’ in‐patient hospital care and cause of death due to alcohol‐related health problems and any of the parents’ disability pensions. Information on parents’ alcohol‐related health problems were extracted from the National Hospital Discharge Register and Cause of Death Register using Sweden's National Board of Health and Welfare index of alcohol‐related morbidity and mortality 33, 34. Lastly, a regional measure of population density of locality was included; this measure is coded from 1 to 10. A locality is a concept used by Statistics Sweden, defined as an urban area with at least 200 inhabitants, where houses are not more than 200 metres apart regardless of municipal or regional boundaries 35. All covariates were categorized as indicated in Table 1.

**Table 1:** Baseline characteristics of the intervention and control group (*n* = 518 810).

	Intervention group (*n* = 70 761) *n* (%)	Control group (*n* = 448 049) *n* (%)	*P*‐value
Sex			
Male	36 677 (51.8)	228 679 (51.0)	< 0.001
Female	34 084 (48.2)	219 370 (49.0)	
Age^a^	17.0 ± 1.7	17.0 ± 1.7	
Country of birth			
Sweden	69 416 (98.1)	438 800 (97.9)	0.004
Outside of Sweden	1345 (1.9)	9249 (2.1)	
Parental level of education^b^			
Primary	42 748 (60.4)	269 481 (60.2)	< 0.001
Secondary	18 559 (26.2)	112 821 (25.2)	
University and above	5098 (7.2)	35 583 (7.9)	
Missing	4356 (6.2)	30 164 (6.7)	
Parental SEI^b^			
High non‐manual	4132 (5.8)	27 752 (6.2)	< 0.001
Middle non‐manual	11 918 (16.8)	78 177 (17.5)	
Low non‐manual	11 584 (16.4)	68 161 (15.2)	
Self‐employed/farmer	5067 (7.2)	40 347 (9.0)	
Skilled workers	20 330 (28.7)	123 239 (27.5)	
Unskilled workers	12 460 (17.6)	74 215 (16.6)	
Others, not classified	4995 (7.1)	34 183 (7.6)	
Missing	275 (0.4)	1975 (0.4)	
Parental disability pension	5959 (8.4)	36 431 (8.1)	0.008
Parental alcohol‐related health problems	5479 (7.7)	29 257 (6.6)	< 0.001
Own highest level of education			
Primary	15 067 (21.3)	92 388 (20.6)	< 0.001
Secondary	33 289 (47.0)	208 994 (46.7)	
University and above	22 152 (31.1)	145 255 (32.4)	
Missing	253 (0.4)	1412 (0.3)	
Population density of locality^c^			
≥ 99 999 inhabitants	33 858 (47.9)	94 662 (21.1)	< 0.001
50 000–99 999 inhabitants	4135 (5.8)	66 844 (14.9)	
20 000–49 999 inhabitants	4592 (6.5)	58 453 (13.1)	
10 000–19 999 inhabitants	3411 (4.8)	47 961 (10.7)	
5000–9999 inhabitants	4428 (6.3)	22 989 (5.1)	
2000–4999 inhabitants	5080 (7.2)	34 532 (7.7)	
1000–1999 inhabitants	1631 (2.3)	18 155 (4.1)	
500–999 inhabitants	1675 (2.4)	13 553 (3.0)	
200–499 inhabitants	1225 (1.7)	11 990 (2.7)	
Area not defined as a locality	10 726 (15.2)	78 910 (17.6)	

SEI = socio‐economic index.

a Mean age (with standard deviation) when the policy change was initiated (1 November 1967).

b Population and Housing Censuses 1970.

c A locality is defined by Statistics Sweden defined as an urban area with at least 200 inhabitants where houses are not more than 200 metres apart, regardless of municipal or regional boundaries.

### Statistical analysis

Pearson's χ^2^ test was used test the descriptive differences between the intervention and control groups. The association between increased alcohol availability and disability pension was estimated using Cox proportional hazards regression analysis to obtain hazard ratios (HR) with 95% confidence intervals (CI). Verification of the proportional hazards assumption was conducted using log–log plots and plots of Schoenfeld residuals. Person‐time was calculated from 1 January 1971 until the date of receiving disability pension, date of migration, date of death or until 31 December 2013, whichever came first. In the regression analysis the individual‐ and family‐level covariates were included as potential confounders. Finally, we used a stratified Cox regression model adjusting for population density of locality, thus allowing for unique baseline hazard for each stratum.

Evidence of interaction between increased alcohol availability and sex, as well as childhood socio‐economic status (SES) and SES in adulthood, defined as parental and own level of education in relation to all outcome variables, was assessed using the post‐estimation Wald test. To tests if age modified the association between increased alcohol availability and disability pension, we also conducted an interaction analysis between increased alcohol availability and age, categorized into three age groups (14–15, 16–17, 18–20 years).

Missing values for the baseline variables were coded as separate categories, as the same conclusions were reached when excluding observations with missing data. All analyses were computed using Stata Statistical Software, release 13.

### Sensitivity analysis

A sensitivity analysis was conducted including the outcome disability pension due to any diagnosis excluding alcohol use disorders and mental disorders. It was expected that if there is a causal effect between increased alcohol availability and disability pension due to alcohol use disorders or mental disorders, then this association should be stronger compared to the association between alcohol availability and disability pension due to any diagnosis excluding alcohol use disorders and mental disorders.

Prior to the policy change the intervention group had, on average, a higher alcohol consumption compared to the control group 36. To explore if this difference could potentially have biased our results, an additional sensitivity analysis on a subsample was conducted by comparing individuals living in Gothenburg county (part of intervention group) with individuals living in a county with known higher alcohol consumption in the control group, in this case Stockholm county.

## Results

Table 1 presents the study population distribution of baseline characteristics. No substantial sex interaction in relation to disability pension due to all‐cause (*P* = 0.082), alcohol use disorders (*P* = 0.918) or mental disorders (*P* = 0.537) was found, thus all analyses were combined for males and females. A higher proportion of parents in the intervention group had disability pension and alcohol‐related health problems compared to the parents in the control group. Individuals residing in the intervention area were, to a greater extent, living in a locality with a high population density compared to the individuals residing in the control area.

During the 43‐year follow‐up a total of 104 475 individuals (19.6%) were granted disability pension, of whom 15 090 (21.3%) individuals were in the intervention group and 89 385 (20.0%) were in the control group (Table 2). Among all granted disability pensions, 2476 (2.4%) were due to alcohol use disorders and 26 699 (25.6%) to mental disorders.

**Table 2:** Descriptive statistics of granted disability pension (*n* = 104 475) from 1971 to 2013 of the intervention and control group.

	Intervention group (*n* = 70 761) *n* (%)	Control group (*n* = 448 049) *n* (%)
All‐cause (total)	15 090 (21.3)	89 385 (20.0)
Alcohol use disorders	436 (0.6)	2040 (0.5)
Mental disorders^a^	4343 (6.1)	22 356 (5.0)

a Excluding alcohol use disorders.

During follow‐up, 4305 (6.1%) individuals in the intervention group and 22 415 (5.0%) individuals in the control group emigrated from Sweden. A total of 30 012 deaths were registered and, of those, 4365 were alcohol‐related, 624 (12.4%) in the intervention group and 3741 (12.1%) in the control group.

### Increased alcohol availability and disability pension

In the crude analysis, adolescents exposed to increased alcohol availability were at an increased risk of being granted disability pension due to all‐cause [HR = 1.09, 95% confidence interval (CI) = 1.07–1.11], alcohol use disorders (HR = 1.39, 95% CI = 1.25–1.54) and mental disorders (HR = 1.26, 95% CI = 1.22–1.30) (Table 3). In the fully adjusted model the ratio remained the same for disability pension due to all‐cause. For disability pension due to alcohol use disorders and mental disorder, these ratios decreased to 1.17 (95% CI = 1.05–1.23) and 1.19 (95% CI = 1.15–1.23), respectively.

**Table 3:** Hazard ratios (HRs) with 95% confidence intervals (CIs) for the associations between increased alcohol availability during adolescence and disability pension due to all‐cause, alcohol use disorders and mental disorders.

	Crude HR (95% CI)	Model 1 HR adjusted (95% CI)	Model 2 HR adjusted (95% CI)	Model 3 HR adjusted (95% CI)	Number of events
All‐cause	1.09 (1.07–1.11)	1.09 (1.07–1.11)	1.09 (1.07–1.11)	1.09 (1.07–1.11)	104 475
Alcohol use disorders	1.39 (1.25–1.54)	1.32 (1.20–1.47)	1.32 (1.19–1.47)	1.17 (1.05–1.30)	2476
Mental disorders^a^	1.26 (1.22–1.30)	1.26 (1.22–1.30)	1.26 (1.22–1.30)	1.19 (1.15–1.23)	26 699

a Excluding alcohol use disorders.

Crude analysis: unadjusted model.

Model 1: Adjusted for sex, year of birth, country of birth, highest level of parental education and SEI and any of the parents’ registered in‐patient care and cause of death due to alcohol‐related health problems and any of the parents’ disability pension.

Model 2: Additional adjustment for own level of education.

Model 3: Additionally stratified for population density of locality.

The results of the interaction analyses found that age, childhood SES or later SES did not modify the association between increased alcohol availability and disability pension due to all‐cause, alcohol use disorders or mental disorders.

### Sensitivity analysis

Results from the sensitivity analysis showed that individuals exposed to increased alcohol availability during adolescence had a 1.05 (95% CI = 1.03–1.07) increased risk of receiving a disability pension due to any diagnosis, excluding alcohol use disorder and mental disorders (Supporting information, Table S1), which is a slightly weaker association compared to disability pension due to all‐cause, alcohol use disorders and mental disorders.

Comparing only the individuals living in Gothenburg county to the individuals living in Stockholm county, the positive associations between increased alcohol availability during adolescence and disability pension due to alcohol use disorders (HR = 1.33, 95% CI = 1.16–1.53) and mental disorders (HR = 1.16, 95% CI = 1.11–1.22) remained, after adjusting for all individual‐, family‐ and regional‐level covariates ( Supporting information, Table S2).

## Discussion

The results of this study, using a natural experimental design with a 43‐year follow‐up, found that an increase in the availability of alcohol in Sweden from 1967 to 1968, selling strong beer in grocery stores with a lower age limit (16 years instead of 21 years), was associated with long‐term health consequences. Adolescents exposed to the increased alcohol availability had an increased risk of receiving disability pension due to all‐causes, alcohol use disorders and mental disorders compared to the general population of the same age not exposed.

These results support and extend further the current literature on the long‐term negative effects of alcohol consumption during adolescence in a number of ways 15, 16, 17, 18. First, supporting previous research on increased alcohol availability, we found that lowering the legal age limit for alcohol purchases can have long‐term alcohol‐related health consequences 2, 5, 6, 7, 8, 9. Secondly, the study provides further evidence that alcohol consumption during adolescence is associated with later health problems 16, 17, 18. In line with previous research on male conscripts, increased alcohol availability during adolescence was found to be associated positively with all‐cause disability pension and disability pension due to alcohol use disorders 19, 20, 21. Contradicting previous research, the current results demonstrated a positive association between increased alcohol availability during adolescence and disability pension due to mental disorders 20. It is difficult, however, to compare the present results with previous research, as the definition of exposure and follow‐up time differed greatly.

Further, as anticipated, the association between increased alcohol availability and disability pension due to any diagnosis excluding alcohol use disorders and mental disorders was weaker compared to the main outcomes of interest. Alcohol consumption is a necessary cause in order to be diagnosed with an alcohol‐related health problem such as alcohol use disorder; however, high alcohol consumption is also a risk factor for more than 200 diseases 37. Consequently, increased alcohol availability during adolescence might also have increased the risk of a vast amount of other health problems, which could explain the slight elevated risk found in the sensitivity analysis. It is difficult to investigate this issue further with the current design, as we do not have information on the individuals’ prior or current alcohol consumption.

Although there is a vast amount of research suggesting that adolescent alcohol consumption is a strong predictor of alcohol problems in adulthood, the underlying mechanisms of the relationship is not understood fully 16, 25. From a life‐course perspective, adolescence can be considered a sensitive time‐period where individuals are more vulnerable to various social and biological exposures, which can have an impact on health later in life 14, 38. However, it remains unclear if early alcohol consumption is causal, a tracked behaviour or merely a marker of vulnerability to alcoholism 14, 17. With the current results, one cannot draw any conclusions as to whether or not adolescent alcohol consumption is a causal factor for becoming a disability pension beneficiary. Previous research on current alcohol consumption during middle age suggests a U‐shaped relationship to risk of all‐cause disability pension; specifically, abstainers and high alcohol consumers are at an increased risk 39, 40, 41. Abstainers who were previous consumers or previous excessive consumers were at a higher risk of disability pension compared to constant abstainers 41. More research on drinking habits in both adolescence and adult life is needed to be able to measure the independent and cumulative effect of alcohol consumption during adolescence on later alcohol‐related health problems.

### Strengths and limitations

Using a population‐based natural experiment with more than 40 years of follow‐up, a large sample size, a restricted time‐window, a well‐defined geographical area and register‐based linkage are major strengths.

Previous research has relied upon self‐reported measures of alcohol consumption, which is highly sensitive to bias due to social desirability 42. Social desirability was not relevant in the current study, as the increased access to alcohol among adolescents was induced exogenously 43. However, the exposure is based on aggregated data with increased alcohol availability used as a proxy for increased alcohol consumption. During the policy experiment, there was a 10‐fold increase of strong beer sales in the intervention area and a 1.26‐fold increase in the control area 24. Furthermore, the experiment terminated early due to increased reports of alcohol‐related problems, especially among youth 29. The lack of individual data on alcohol consumption, however, means that the role of adolescents’ drinking patterns in relation to all‐cause and cause‐specific disability pension could not be examined.

The outcome was collected from high‐quality and reliable registers, eliminating the risk of recall bias and bias due to attrition. The register also includes information on the underlying diagnosis of the disability pension, which adds valuable information. A limitation is, however, that the register included all disability pensioners from 1 January 1971 who are still alive and receiving disability pension from 1994. Consequently, we excluded all individuals who died before 1994, as they were not part of the register. This was to reduce the possibility of introducing bias into our estimates, as we do not know if these individuals received any disability pension. Analysis including the excluded individuals and analysis starting follow‐up in 1994 showed the same results as the results presented here. We estimate that approximately 2% of all disability pension cases were missed because of this truncation in the register. Further, the register allows only three levels of ICD codes to specify the diagnosis of the disability pension. Consequently, as the ICD‐8 and ICD‐9 requires four levels to specify that the diagnosis is alcohol‐related, we were unable to include more alcohol‐related diagnoses. Although the legislation in Sweden changed in 1977, with regard to receiving disability pension due to alcohol abuse it is important to acknowledge that the role of alcohol consumption might be overlooked for a more ‘socially acceptable’ diagnosis 20, 44. Thus, there could be a degree of misclassification on the underlying diagnosis of the disability pension, which could have attenuated the effect of increased alcohol availability in adolescence on disability pension due to alcohol use disorders.

Due to data constraints, we were unable to compare the present results with a time‐period before and after the alcohol policy change. A comparison of this kind could deal with any potential selection bias that might affect the response to the alcohol policy change 43. According to reports on Alcohol Statistics in Sweden from 1965 registered sales statistics, the counties belonging to the intervention group had, on average, slightly higher strong beer consumption, 0.5 litres of strong beer per capita per year, compared to the counties belonging to the control group before the alcohol policy change in 1967 36. However, the results of the sensitivity analysis demonstrated similar effects of the alcohol policy changes, suggesting that although prior intrinsic differences in alcohol consumption might bias our results slightly, the positive association between increased alcohol availability and risk of receipt of all‐cause and cause‐specific disability pension remained.

## Conclusion

Using results from a natural experiment, this study showed that increased alcohol availability during adolescence, as a result of the alcohol policy change in Sweden during 1967/1968, was associated with an increased risk of receiving disability pension due to all‐cause, alcohol use disorders and mental disorders later in life. Although alcohol norms and alcohol policy differ greatly between countries, this study demonstrated additional evidence of the importance of having an alcohol policy that restricts the availability of alcohol among adolescents to decrease the harm related to alcohol.

## Declaration of interests

None.

## Supporting information


**Table S1** Hazard ratios (HRs) with 95% confidence intervals (CIs) for the associations between increased alcohol availability during adolescence and disability pension due to all‐cause, alcohol use disorders, mental disorders and any diagnosis.
**Table S2** Results from the additional sensitivity analysis comparing individuals from Gothenburg county (intervention group) and Stockholm county (control group). Hazard ratios (HRs) with 95% confidence intervals (CIs) for the associations between increased alcohol availability during adolescence and disability pension due to all‐cause, alcohol use disorders and mental disorders.
