# Supplementary material for: Effects of increased alcohol availability during adolescence on the risk of all‐cause and cause‐specific disability pension: a natural experiment
Source: Addiction. 2017 Feb 7;112(6):1004–12. doi: 10.1111/add.13750 (PMC5434812; doi:10.1111/add.13750)
Supplement: Supplementary file 1 — Table S1 Hazard ratios (HRs) with 95% confidence intervals (CIs) for the associations between increased alcohol availability during adolescence and disability pension due to all‐cause, alcohol use disorders, mental disorders and any diagnosis. Table S2 Results from the additional sensitivity analysis comparing individuals from Gothenburg county (intervention group) and Stockholm county (control group). Hazard ratios (HRs) with 95% confidence intervals (CIs) for the associations between increased alcohol availability during adolescence and disability pension due to all‐cause, alcohol use disorders and mental disorders. [file ADD-112-1004-s001.docx]

Supplementary Table 1: Hazard ratios (HRs) with 95% confidence intervals (CIs) for the associations between increased alcohol availability during adolescence and disability pension due to all-cause, alcohol use disorders, mental disorders and any diagnosis.

|  | Crude HR  (95%CI) | Model 1  HR adjusted  (95%CI) | Model 2  HR adjusted  (95%CI) | Model 3  HR adjusted  (95%CI) | Number of events |
| --- | --- | --- | --- | --- | --- |
| All-cause | 1.09 (1.07-1.11) | 1.09 (1.07-1.11) | 1.09 (1.07-1.11) | 1.09 (1.07-1.11) | 104 475 |
| Alcohol use disorders | 1.39 (1.25-1.54) | 1.32 (1.20-1.47) | 1.32 (1.19-1.47) | 1.17 (1.05-1.30) | 2476 |
| Mental disorders^1^ | 1.26 (1.22-1.30) | 1.26 (1.22-1.30) | 1.26 (1.22-1.30) | 1.19 (1.15-1.23) | 26 699 |
| Any diagnosis^2^ | 1.03 (1.01-1.05) | 1.03 (1.01-1.05) | 1.03 (1.00-1.05) | 1.05 (1.03-1.07) | 76 286 |

^1^Excluding alcohol use disorders

^2^Excluding alcohol use disorders and mental disorders

Crude analysis: unadjusted model

Model 1: Adjusted for sex, year of birth, country of birth, highest level of parental education and SEI and any of the parent’s registered inpatient care and cause of death due to alcohol-related health problems and any of the parent’s disability pension

Model 2: Additional adjustment for own level of education

Model 3: Additionally stratified for population density of locality

Supplementary Table 2: Results from the additional sensitivity analysis comparing individuals from Gothenburg county (intervention group) and Stockholm county (control group). Hazard ratios (HRs) with 95% confidence intervals (CIs) for the associations between increased alcohol availability during adolescence and disability pension due to all-cause, alcohol use disorders and mental disorders.

|  | Including full analytical sample  *n*= 518 810 | | Including only Gothenburg and Stockholm  *n*= 146 413 | |
| --- | --- | --- | --- | --- |
|  | Adjusted HR  (95%CI) | Number of events | Adjusted HR  (95%CI) | Number of events |
| All-cause | 1.09 (1.07-1.11) | 104 475 | 1.13 (1.10-1.16) | 28 616 |
| Alcohol use disorders | 1.17 (1.05-1.30) | 2476 | 1.33 (1.16-1.53) | 893 |
| Mental disorders^1^ | 1.19 (1.15-1.23) | 26 699 | 1.16 (1.11-1.22) | 9194 |

^1^Excluding alcohol use disorders

HR adjusted for sex, year of birth, country of birth, highest level of parental education and SEI and any of the parents' registered inpatient care and cause of death due to alcohol-related health problems, any of the parents’ disability pensions, own level of education and stratified for population density of locality
